# Reported History of Measles and Long-term Impact on Tetanus Antibody Detected in Children 9–59 Months of Age and Receiving 3 Doses of Tetanus Vaccine in the Democratic Republic of the Congo

**DOI:** 10.1097/INF.0000000000003840

**Published:** 2023-02-09

**Authors:** Hayley R. Ashbaugh, James D. Cherry, Nicole A. Hoff, Reena H. Doshi, Patrick Mukadi, Stephen G. Higgins, Roger Budd, Christina Randall, Emile Okitolonda-Wemakoy, Jean Jacques Muyembe-Tamfum, Sue K. Gerber, Christine Wells, Anne W. Rimoin

**Affiliations:** From the * Department of Epidemiology, Fielding School of Public Health, University of California, Los Angeles, Los Angeles, California; † David Geffen School of Medicine at UCLA, Los Angeles, California; ‡ Kinshasa University, School of Medicine, Kinshasa, Democratic Republic of the Congo; § Personal Genome Diagnostics, Baltimore, Maryland; ¶ DYNEX Technologies Incorporated, Chantilly, Virginia; ‖ National Institute for Biomedical Research, Kinshasa, Democratic Republic of the Congo; ** Bill and Melinda Gates Foundation, Seattle, Washington; †† UCLA IDRE Statistical Consulting Group, University of California, Los Angeles, Los Angeles, California.

**Keywords:** antibody response, Democratic Republic of the Congo, measles, tetanus

## Abstract

Recent studies suggest measles-induced immune amnesia could have long-term immunosuppressive effects via preferential depletion of memory CD150+ lymphocytes, and associations with a 2–3 year period of increased mortality and morbidity from infectious diseases other than measles has been shown in children from wealthy and low-income countries. To further examine the associations previous measles virus infection may have on immunologic memory among children in the Democratic Republic of the Congo (DRC), we assessed tetanus antibody levels among fully vaccinated children, with and without a history of measles.

**Methods:**

We assessed 711 children 9–59 months of age whose mothers were selected for interview in the 2013–2014 DRC Demographic and Health Survey. History of measles was obtained by maternal report and classification of children who had measles in the past was completed using maternal recall and measles IgG serostatus obtained from a multiplex chemiluminescent automated immunoassay dried blood spot analysis. Tetanus IgG antibody serostatus was similarly obtained. A logistic regression model was used to identify association of measles and other predictors with subprotective tetanus IgG antibody.

**Results:**

Subprotective geometric mean concentration tetanus IgG antibody values were seen among fully vaccinated children 9–59 months of age, who had a history of measles. Controlling for potential confounding variables, children classified as measles cases were less likely to have seroprotective tetanus toxoid antibody (odds ratio: 0.21; 95% confidence interval: 0.08–0.55) compared with children who had not had measles.

**Conclusions:**

History of measles was associated with subprotective tetanus antibody among this sample of children in the DRC who were 9–59 months of age and fully vaccinated against tetanus.

Recent studies suggest a measles-induced immune amnesia that could have long-term immunosuppressive effects via preferential depletion of memory CD150+ lymphocytes.^1–3^ An association with a 2–3-year period of increased mortality and morbidity from infectious diseases other than measles has been shown in children from wealthy countries,^4–6^ and increased morbidity of clinical signs of infectious disease was shown in children 9–59 months of age in the Democratic Republic of the Congo (DRC) who had been classified as measles cases within the past 2 years.^7^

Vaccine-induced immunologic memory, as with natural immunity, is mediated by B and T memory cells. While immunity against many pathogens, such as measles virus, can be achieved either via natural infection or immunization, this is not the case with tetanus. Tetanus, a disease caused by a neurotoxin from the bacterium *Clostridium tetani*, can be prevented via immunization, but natural infection with tetani toxin will not result in protective antibody against future infection.^8,9^ Three doses of tetanus toxoid are thought to confer protection against tetanus for at least 5 years, and the accepted minimal level of toxoid-induced antitoxin antibody required to protect against disease is 0.01 IU/mL, as measured via the “gold standard” in vivo neutralization assay.^8^ While tetanus is uncommon in wealthy countries, it is a greater concern in middle- and low-income countries, particularly those whose healthcare delivery is disrupted by armed conflict, with 79% of deaths due to tetanus estimated to occur in south Asia and sub-Saharan Africa (44,612 and 56,743, respectively).^9,10^

In 2015, there was a mortality rate of 1.34 per 100,000(confidence interval [CI]: 0.24-6.35) due to non-neonatal tetanus in the DRC.^10^ While this is an approximately 50% decrease from the 1990 mortality estimate, it is still too high for this vaccine-preventable disease. In 2018 and 2019, the DRC had a reported incidence rate (IR) for total tetanus (including neonatal tetanus) of 9.4 and 9.3, respectively. By comparison, the African Region had IRs of 5 and 3.7 for the same years, and the global IRs were 3.1 and 2.7 for 2018 and 2019, respectively. DRCs total tetanus IR for 2020 and 2021 was only 0.5,^11^ but this may have been due to limited surveillance during the severe acute respiratory syndrome coronavirus 2 pandemic. Immunization against tetanus in DRC is recommended at 6, 10 and 14 weeks via the Diphtheria and Tetanus and Pertussis and *Haemophilus influenzae* and Hepatitis B vaccine (DTwPHibHepB),^11^ yet findings from the 2013-2014 DRC Demographic and Health Survey (DHS) reveal that only 60% of children 6–59 months of age received the full 3-dose tetanus toxoid-containing series, and only 40% had seroprotective antibody titres.^12^

To further examine the associations previous measles virus infection may have on immunologic memory among children in the DRC, we examined tetanus antitoxin levels among children fully vaccinated against tetanus, with and without a history of measles. Because natural infection with tetanus toxin does not induce seroprotection, tetanus antibody is ideal for assessment of measles virus’ potential impact on vaccine-induced immunologic memory in this population. Previously, we presented a statistically significant difference in vaccine-induced immunity to tetanus among children with past measles disease versus children who had not had measles.^13^ Here, we provide additional analyses assessing this association and include examination of seroprotective tetanus antibody levels as an additional endpoint.

## METHODS

We examined the association between past measles cases and tetanus antibody levels among children participating in the 2013–2014 DRC DHS. Our sample consisted of 711 children 9–59 months old.

### Data Source and Study Population

The 2013–2014 DHS, a nationally representative survey based on a stratified 2-stage cluster design, occurred from November 2013 to February 2014. The first stage of the survey consisted of Enumeration Area formation in which a stratified sample of geographic locations, or clusters (n = 540), was selected with proportional probability according to size. The second stage involved sampling households from each Enumeration Area; complete listings of households were created within each cluster, and households (n = 9000) were selected with equal probability.^14–17^ Within the selected households, individuals interviewed included 18,827 women 15–49 years old (all selected households) and 8656 men 15–59 years old (50% of selected households). The DHS collected biomarker data only on children from households in which men were interviewed. In our dataset, there were 812 children 9–59 months old with written (and dated) record of receiving the full 3-dose tetanus vaccine series and who also had anthropometric, measles and tetanus IgG serology data available, and of these, 713 children had data on measles that occurred following DTwPHibHepB vaccination as well as all other covariates of interest. Four of these children who met measles case criteria had had measles less than 2 months before dried blood spot (DBS) collection and so were removed from analyses to prevent associations due to acute measles-induced immune suppression in this study, bringing the final total to 711 children (this total incorporates complex survey weighting methods utilized by the 2013–2014 DRC DHS).

Information was collected on weight, health outcomes, vaccination history and vaccine-preventable disease serology. After parental consent, DBSs were collected from participants to assess immunity to vaccine-preventable diseases and processed at the University of California, Los Angeles (UCLA)—DRC laboratory at the National Laboratory for Biomedical Research in Kinshasa. All survey data were transferred from paper questionnaires to an electronic format using the Census and Survey Processing System (U.S. Census Bureau, ICF Macro). Data were double entered and verified by comparison of both datasets.

At the time of interview, if mothers possessed a health care worker-provided vaccination card indicating the date the child was vaccinated, this was considered a “dated card” report. Since “dated card” reports are considered most reliable, we utilized only this type of report when categorizing a child as vaccinated, whether the vaccination was against tetanus or measles. Children classified as “unvaccinated” in this study were those reported as such in the 2013–2014 DHS. Tetanus vaccination status was obtained via dated vaccination card and limited to children receiving the complete 3-dose vaccination series (all other children were removed from analyses). Due to concern of live versus killed vaccines resulting in nonspecific effects on vaccinees,^18^ we examined whether the DTwPHibHepB versus measles vaccine was given last within the sample of participating children. Of the 649 children who received both DTwPHibHepB and measles vaccines, 5 (0.1%) children received DTwPHibHepB after the measles vaccine (indicating an incorrect vaccination schedule), 22 (3.3%) children received both vaccines within the same month and 622 (96%) received the measles vaccine after DTwPHibHepB. We did not examine other vaccines a child may have received.

### Laboratory Analysis

DBS samples were extracted using a modified extraction protocol^19^ and processed at the UCLA-DRC laboratory at the National Laboratory for Biomedical Research. The DBS extraction and assay protocol and multiplex technology have been described elsewhere.^20,21^ Briefly, a 0.25“ DBS punch was extracted, shaking at room temperature, in 1 mL phosphate buffered saline, 0.05% Tween-20 and 5% dried milk, which represents a 1:143-fold dilution assuming 7 µL of serum per punch. Polystyrene beads coated separately with antigen were immobilized within 54-well SmartPLEX assay strips with 10 beads per well and processed using a prototype DYNEX Multiplier chemiluminescent automated immunoassay platform with a research use only Smart-PLEX assay for measles, mumps, rubella, varicella-zoster virus and tetanus (DYNEX Technologies, Chantilly, VA).^21^ Assay Score (AS) for each sample was calculated as a ratio of the signal to a 5-donor, pooled positive control included in each run. Given that it was outside the scope of this project, this research use only immunoassay panel was not correlated with International Units for measles antibody via the plaque reduction neutralization test. In previous work,^22^ we have reported antibody according to the AS, but in an effort to keep units consistent in this study, we converted the measles AS to IU/mL. For tetanus IgG antibody, based on validation (R. Budd, personal communication, 15 January 2020) and epidemiologic studies,^8^ the seroprotective cutoff was set to a value of 0.69 AS, which corresponds to 0.2 IU/mL.

As part of our measles case definition, we utilized the limit of quantitation (LOQ) for measles antibody, which was calculated to be 12.06 IU/mL, and this value, in supplement to maternal report of measles, was used as an additional inclusion criterion for classifying previous measles disease among children with maternally reported history of measles. Maternal report captured whether the child had ever had measles in the past, and most maternal reports of measles had an associated month and year date. Requiring maternally reported measles cases to have a minimum measles antibody level to be included as measles cases in this analysis was done to decrease misclassification of maternal reports of measles cases, yet avoid missing cases of infants who may not have displayed a robust immune response despite previous infection. Among the measles-vaccinated children (n = 649), 42 children had a maternal report of measles. Of these 42, 40 (95%) met the LOQ antibody threshold while 2 (5%) were below this threshold and so excluded as measles cases (coding them as noncases for measles). Among children not vaccinated against measles (n = 63), 2 children had a maternal report of measles, and of these, 2 (100%) met the LOQ antibody threshold. To further examine the possibility of misclassification of measles, we included a sensitivity analysis, described in the statistical analysis section.

### Covariate Selection

A priori confounders, such as age, sex and geographic location, were included in descriptive and regression analyses. Other potential confounding variables were identified in the literature and based largely on variables representing social determinants of health and found by others to capture health disparities, such as chronic malnutrition (stunting), maternal education, wealth index and birth order.^16,23,24^ Malaria status (via blood smear) was included due to its association with host immunosuppression,^25^ and the authors previously found that rural versus urban residence impacted measles vaccination coverage.^26^ Finally, due to the history of conflict in the DRC, we created a dichotomous variable of children living in provinces experiencing high levels of conflict versus all other provinces. The 5 (old) provinces (Katanga, Maniema, Nord-Kivu, Orientale and Sud-Kivu) with the highest levels of human displacement in 2014 were classified as provinces experiencing high levels of conflict, as most of the human displacement (97%) was conflict-related.^27,28^

Due to the challenges inherent with directly measuring a household’s wealth, the DHS assumes that the economic state of a household is related to whether the household has access to certain amenities or services. Such variables can easily be included into a questionnaire or directly observed by the interviewer. Within the DHS, households are placed into quintiles according to household ownership of previously selected assets and via principal components analysis. Asset scores are standardized relative to a standard normal distribution and each household receives a summed score based on the standardized score for each asset. Individuals receive a score reflective of their household score, and the resulting quintiles make up the wealth index for the DHS survey.^29^ In this study, we dichotomized wealth index to compare the most disadvantaged individuals to all others in the sample, resulting in a wealth index that compares the poorest children to all others, or the lowest quintile children to those in the combined group of second lowest, middle, fourth lowest and highest quintile.

Collinearity diagnostic statistics were performed on all explanatory variables included in the final regression models, with acceptable results (tolerance for all variables was ≥ 0.75, variance inflation factor for all variables was ≤ 1.33) for all variables of interest.

### Statistical Analyses

Descriptive statistics were performed, examining associations of sociodemographic covariates with both tetanus antibody seroprotection and measles case status via Wald χ^2^ test. Because our sample was limited to children with 3 doses of DTwPHibHepB reported via dated vaccination card, we observed some differences in descriptive data for variables such as wealth index, rural versus urban residence and measles vaccination status, relative to the authors’ previous work examining these variables among children with vaccinations reported via maternal recall or marked card. One such difference was that the overall measles attack rate (AR) was higher for children who were measles vaccinated versus unvaccinated in this analysis. To further explore this finding, we provided an additional table stratifying AR by age and time of initial measles vaccination, as the subgroup ARs are more reflective of findings from the larger sample of children in DRC including those not fully vaccinated against tetanus.^22^

Multiple linear regression was used to examine the linear relationship between tetanus antibody and history of measles, adjusting for potential confounding variables. We examined the relationship between our log transformed continuous tetanus antibody variable and measles case status, adjusting for covariates. Multiple logistic regression was used to examine predictors of tetanus seroprotection among this fully vaccinated group of children. Additionally, we examined differences in antibody among subgroups by calculating geometric mean concentration (GMC) of tetanus IgG by age, measles status and other covariates of interest. To control for variability in probability of selection and interview, DHS stratum, cluster and individual sampling weights were applied to analyses and SAS 9.4 PROC SURVEY procedures were utilized.

Because laboratory confirmation of maternally reported measles cases was beyond the scope of this study, to examine the potential effects of misclassification of measles cases (such as incorrectly classifying a child as being a measles case in the past when in fact the child had not been a measles case), we performed additional analyses to examine GMC of tetanus antibody by subgroup. A limitation in categorizing a measles case via positive maternal + LOQ is that children who have been measles vaccinated are more likely than unvaccinated children to meet LOQ criterion even if the maternal report is falsely positive. We have no way of differentiating whether children who meet the LOQ criterion do so due to vaccination or prior infection, but as a mitigating measure, we have provided a closer examination of measles antibody among the following 4 groups of children: (1) measles-unvaccinated + no maternal measles report, (2) measles-unvaccinated + maternal measles report, (3) measles-vaccinated + no maternal measles report and (4) measles-vaccinated + maternal measles report.

To examine the impact that varying levels of misclassification might have on odds ratio (OR) estimates in our logistic regression model, we conducted a supplementary sensitivity analysis. Our concern was that we might be more likely to include false positive measles cases (children categorized as having had measles in the past due to positive maternal report and qualifying LOQ that were in fact not measles cases) among children who were vaccinated against measles versus children who had not received measles vaccine. While the authors think it is plausible that probability of misclassification of measles case status (exposure) is independent of probability of misclassification of tetanus seroprotection status (outcome), due to tetanus seroprotection status being measured via biosample, there is concern of nondifferential dependent misclassification between measles case status (exposure) and measles vaccination (covariate).^30^

Utilizing unweighted data, we demonstrated via 2 × 2 tables the potential impact of nondifferential dependent misclassification between measles case status and measles vaccination, with 10%, 25% and 50% of measles cases among vaccinated children considered to be false positives and so reclassified as measles noncases. Using updated values for exposure (measles status) and outcome (tetanus seroprotection status) variables, we acquired ORs and 95% Wald confidence limits via univariate regression.

All analyses were performed using SAS software, Version 9.4 (SAS Institute, Cary, NC). Ethical approval was obtained at UCLA Fielding School of Public Health, the Kinshasa School of Public Health and the U.S. Centers for Disease Control and Prevention. As children were younger than the standard age of assent, the parent or guardian of participating children provided consent on the child’s behalf.

## RESULTS

### Descriptive Analyses

Descriptive statistics (Table 1) show that, among children receiving 3 doses of tetanus vaccine, seroprotection for tetanus varied by age (Wald χ^2^ *P* = 0.0057), maternal education, wealth index and severe stunting. Measles case status varied by age (Wald χ^2^ *P* = 0.0253) and wealth index (Wald χ^2^ *P* = 0.0111).

**TABLE 1.** Descriptive Data in Children 9–59 Months of Age, by Tetanus Seroprotective Status and Measles Report (n = 711)

Variable	Total	Seroprotection for Tetanus*		Measles Case	
	n	n	%	n	%
Age (months)					
9–11	75	31	41	1	1
12–23	233	167	72	4	2
24–35	179	109	61	7	4
36–47	105	53	50	16	15
48–59	119	60	50	14	12
*P* †		**0.0057**		**0.0253**	
Sex					
Male	330	195	59	22	7
Female	381	226	59	20	5
*P*		0.9682		0.5104	
Measles vaccination					
Yes	649	393	61	40	6
No	63	28	44	2	3
*P*		0.0821		0.4815	
Maternal education					
< 7 years	352	181	51	23	7
≥ 7 years	360	240	67	19	5
*P*		**0.003**		0.6118	
Wealth index‡					
Poorest	75	30	40	1	1
All others	636	391	61	41	6
*P*		**0.0052**		**0.0111**	
Rural vs. urban residence					
Urban	347	227	65	19	5
Rural	365	194	53	23	6
*P*		0.0584		0.7667	
Severely stunted§					
Yes	133	57	43	8	6
No	579	364	63	34	6
*P*		**0.0032**		0.9599	
Firstborn vs. later born					
Firstborn	186	124	67	12	6
Later born	525	297	57	31	6
*P*		0.097		0.8538	
Malaria (blood smear) result					
Positive	82	44	54	7	9
Negative	630	377	60	35	6
*P*		0.3046		0.5604	
Conflict					
High conflict province¶	307	171	56	22	7
Other province	405	250	62	21	5
*P*		0.3419		0.4485	
Province					
Kinshasa	109	78	72	4	3
Bandundu	60	29	48	4	7
Bas-Congo	43	24	56	0	1
Equateur	70	42	60	6	9
Kasai-Occidental	78	47	60	1	1
Kasai-Oriental	46	29	63	5	11
Katanga	46	27	59	4	9
Maniema	6	5	83	—	—
Nord-Kivu	170	91	54	14	8
Orientale	13	7	54	1	8
Sud-Kivu	72	43	60	2	3
*P*		0.5588		—	
Total	**711**	**421**	**59**	**42**	**6**

Bold entries indicate statistically significant estimates via the Wald X2 test.

The — indicates that no measles cases were reported.

* Seroprotection defined as 0.2 IU/mL.

† Wald χ^2^ test for independence of measles status and row variables.

‡ Wealth index is the Demographic and Health Survey composite measure of a household’s cumulative living standard. Based on household ownership of previously selected assets, and utilizing principal components analysis, households are placed within 1 of 5 quintiles. The dichotomized variable compares the poorest children to all others, or the lowest quintile children to those in the combined group of second lowest, middle, fourth lowest and highest quintile.

§ Calculated according to National Center for Health Statistics/CDC/WHO international reference standard for height/age, dichotomized as –2.0 to ≤ –3.0 SD below the mean (chronically malnourished, or stunted) and normal to ≥ 3.0 SD above the mean (normal/overnourished). Here, we compare the most severely stunted children with all others.

¶ Dichotomous variable of children living in provinces experiencing high levels of conflict (Katanga, Maniema, Nord-Kivu, Orientale and Sud-Kivu) vs. all other provinces. Classified according to the United Nations Office for the Coordination of Humanitarian Affairs.

CDC indicates Centers for Disease Control and Prevention; WHO, World Health Organization.

Examination of measles AR stratified by age (Table 2) revealed among both vaccinated and unvaccinated children, most measles reports occurred during the 24–59 months of age range. Most unvaccinated children fell within the 9–11 and 12–23 months of age groups (n = 53); there were few measles reports (n = 5) overall in these age groups, with none being in unvaccinated children. Only 10 unvaccinated children were in the 24–59 months of age group. The overall AR among the vaccinated (AR_v_) was 6.2 and among the unvaccinated (AR_U_), 3.2. Among the 34 vaccinated children 24–59 months of age in our study who reported measles, 32 (94%) received measles vaccination at less than 12 months of age. There were no cases of measles among unvaccinated children in the 9–11 month and 12–23 months of age groups, and the AR_U_ findings within the 24–59 months of age group was 20.0.

**TABLE 2.** Measles Attack Rate Stratified by Age in Children 9–59 Months of Age (n = 711)

Age (Months)	No History of Measles	Measles Case	Total (n)	Attack Rate (per 100)*,†
9–11				
Unvaccinated	26	0	26	0.0
Vaccinated	48	1	49	2.0
12–23				
Unvaccinated	27	0	27	0.0
Vaccinated	202	4	206	1.9
< 12 months of age‡	182	4	186	2.2
≥ 12 months of age	20	0		
24–59				
Unvaccinated	8	2	10	20.0
Vaccinated	359	34	393	8.7
< 12 months of age	308	32		
≥ 12 months of age	51	2		
Total				
Unvaccinated	60	2	62	3.2
Vaccinated	609	40	649	6.2

* Attack rate in the unvaccinated calculated as: (cases among the unvaccinated/total unvaccinated) × 100.

† Attack rate in the vaccinated calculated as (cases among the vaccinated/total vaccinated) × 100.

‡ Denotes age at which the child was vaccinated against measles.

### Regression Analyses

A linear regression model (Table 3) was used to examine relationship between tetanus antibody and measles and other covariates and revealed statistically significant relationships between age in months, measles case status and wealth index and tetanus antibody. Notably, measles case status predicted a 37% decrease in tetanus antibody and being wealthier (in any wealth category above “poorest”) predicted an expected 67% increase in tetanus antibody. Measles vaccination did not demonstrate a statistically significant relationship with tetanus antibody but showed a 39% increase (not statistically significant) in tetanus antibody among vaccinated children.

**TABLE 3.** Linear Regression of Predictors of Tetanus Antibody Among Fully Vaccinated Children 9–59 Months of Age (n = 711)

Variable	Univariate Regression Coefficients and 95% CI	Multiple Regression Coefficients and 95% CI
Age, months	–0.01 (–0.02 to –0.002)	**–0.01 (–0.02 to –0.001**)
Sex		
Male vs. female (reference)	0.10 (–0.10 to 0.30)	0.12 (–0.09 to 0.32)
Measles case status		
Case vs. noncase (reference)	**–0.54 (–0.94 to –0.15**)	**–0.46 (–0.84 to –0.08**)
Measles vaccination		
Vaccinated vs. unvaccinated (reference)	0.21 (–0.26 to 0.68)	0.33 (–0.09 to 0.76)
Maternal education		
≥ 7 years vs. < 7 years (reference)	**0.30 (0.05–0.56**)	0.03 (–0.22 to 0.29)
Birth order		
Firstborn vs. later born (reference)	0.13 (–0.08 to 0.34)	0.06 (–0.13 to 0.26)
Malnutrition*		
Severely stunted vs. all others (reference)	**–0.34 (–0.57 to –0.11**)	–0.12 (–0.35 to 0.11)
Malaria status		
Positive vs. negative (reference)	–0.19 (–0.43 to 0.05)	–0.15 (–0.42 to 0.11)
Residence		
Urban vs. rural (reference)	**0.32 (0.03–0.62**)	0.16 (–0.17 to 0.48)
Wealth index†		
Wealthier vs. poorest (reference)	**0.56 (0.25–0.86**)	**0.51 (0.17–0.85**)
Conflict‡		
Conflict area vs. nonconflict (reference)	–0.18 (–0.46 to 0.11)	–0.12 (–0.42 to 0.17)

* Calculated according to National Center for Health Statistics/CDC/WHO international reference standard for height/age, dichotomized as –2.0 to ≤ –3.0 SD below the mean (chronically malnourished, or stunted) and normal to ≥ 3.0 SD above the mean (normal/overnourished). Here, we compare the most severely stunted children with all others.

† Wealth index is the Demographic and Health Survey composite measure of a household’s cumulative living standard. Based on household ownership of previously selected assets, and utilizing principal components analysis, households are placed within 1 of 5 quintiles. The dichotomized variable compares the poorest children to all others, or the lowest quintile children to those in the combined group of second lowest, middle, fourth lowest and highest quintile.

‡ Dichotomous variable of children living in provinces experiencing high levels of conflict (Katanga, Maniema, Nord-Kivu, Orientale and Sud-Kivu) vs. all other provinces. Classified according to the United Nations Office for the Coordination of Humanitarian Affairs.

CDC indicates Centers for Disease Control and Prevention; WHO, World Health Organization.

A logistic regression model (Table 4) was used to examine the association between measles case status and tetanus seroprotection. Controlling for potential confounding variables, children with past measles were less likely to have tetanus antibody that reached a seroprotective level (OR: 0.21; 95% CI: 0.08–0.55) compared with children without a history of measles. Measles vaccination itself showed an association with increased seroprotection (OR: 2.65; 95% CI: 1.23–5.68), while severe stunting showed a negative association (OR: 0.55; 95% CI: 0.32–0.96) with tetanus antibody seroprotection in both crude and adjusted models. Not being among the poorest children according to wealth index showed a strong association with tetanus seroprotection (OR: 2.15; 95% CI: 1.19–3.91).

**TABLE 4.** Predictors of Tetanus Seroprotection Among Fully Vaccinated Children 9–59 Months of Age (n = 711)

Variable	Crude Odds Ratio and 95% CI	Adjusted Odds Ratio* and 95% CI
Age, months	0.99 (0.97–1.00)	0.99 (0.98–1.01)
Sex		
Male vs. female (reference)	1.01 (0.71–1.44)	1.01 (0.69–1.48)
Measles case status		
Case vs. noncase (reference)	**0.22 (0.08–0.59**)	**0.21 (0.08–0.55**)
Measles vaccination		
Vaccinated vs. unvaccinated (reference)	1.97 (0.95–4.06)	**2.65 (1.23–5.68**)
Maternal education		
≥ 7 years vs. < 7 years (reference)	**1.87 (1.25–2.80**)	1.25 (0.77–2.04)
Birth order		
Firstborn vs. later born (reference)	1.53 (0.94–2.52)	1.36 (0.83–2.21)
Malnutrition*		
Severe stunting vs. all others (reference)	**0.45 (0.28–0.74**)	**0.55 (0.32–0.96**)
Malaria status		
Positive vs. negative (reference)	0.77 (0.48–1.25)	0.86 (0.49–1.51)
Residence		
Urban vs. rural (reference)	1.66 (0.99–2.79)	1.07 (0.56–2.06)
Wealth index†		
Wealthier vs. poorest (reference)	**2.36 (1.35–4.12**)	**2.15 (1.19–3.91**)
Conflict‡		
Conflict area vs. nonconflict (reference)	0.78 (0.47–1.30)	0.89 (0.48–1.64)

* Calculated according to National Center for Health Statistics/CDC/WHO international reference standard for height/age, dichotomized as –2.0 to ≤ –3.0 SD below the mean (chronically malnourished, or stunted) and normal to ≥ 3.0 SD above the mean (normal/overnourished). Here, we compare the most severely stunted children with all others.

† Wealth index is the Demographic and Health Survey composite measure of a household’s cumulative living standard. Based on household ownership of previously selected assets, and utilizing principal components analysis, households are placed within 1 of 5 quintiles. The dichotomized variable compares the poorest children to all others, or the lowest quintile children to those in the combined group of second lowest, middle, fourth lowest and highest quintile.

‡ Dichotomous variable of children living in provinces experiencing high levels of conflict (Katanga, Maniema, Nord-Kivu, Orientale and Sud-Kivu) vs. all other provinces. Classified according to the United Nations Office for the Coordination of Humanitarian Affairs.

CDC indicates Centers for Disease Control and Prevention; WHO, World Health Organization.

### Geometric Mean Concentrations of Tetanus Antibody

Among the entire sample of 711 children (Supplementary Table 1, http://links.lww.com/INF/E925), tetanus antibody GMC was 0.257 (95% CI: 0.221–0.298), with measles cases (n = 42) having a GMC of 0.155 (95% CI: 0.103–0.232) and children with no history of measles (n = 669) having a GMC of 0.265 (95% CI: 0.228–0.309). Statistically significant differences in GMC were seen between measles cases and noncases (mean difference: –0.540 [0.201]; *P* = 0.0074), as well as urban versus rural children, those with more versus less educated mothers, the most severely malnourished versus all others, and notably, the poorest versus wealthier children (mean difference: –0.555 [0.154]; *P* = 0.0003). In addition to children who had been measles cases, subprotective GMC values were seen among the poorest children (0.156; 95% CI: 0.119–0.205) and those who were severely stunted (0.195; 95% CI: 0.156–0.242).

### Sensitivity Analysis

Examining LOQ, maternal report of measles, measles seroprotection status and GMC of tetanus antibody among subgroups of (1) measles-unvaccinated + no maternal measles report, (2) measles-unvaccinated + maternal measles report, (3) measles-vaccinated + no maternal measles report and (4) measles-vaccinated + maternal measles report (Supplementary Table 2, http://links.lww.com/INF/E926), revealed differences in GMC. Among the measles-unvaccinated, there was a mean difference of 3.64 (95% CI: 2.98–4.31), comparing measles antibody GMC between children with and without maternal report of measles. However, this group only had 2 children with maternal report of measles, so this must be taken into consideration. Among the measles-vaccinated, there was a mean difference of 0.3 (–0.1802 to 0.8528), not statistically significant. This is not surprising, considering all children should have measles antibody from vaccination. Among children with no maternal measles report, there was a mean difference of 1.52 (0.92–2.12) comparing measles antibody between children with and without vaccination (data not shown). Although the group of 62 children with no measles vaccination and no maternal report of measles, surprisingly, had an overall GMC of 0.054 (0.03–0.096), and this likely indicates either misclassification of measles vaccination status or measles disease status, the GMC is below the seroprotective threshold.

Examining exposure misclassification by measles vaccination status (Supplementary Table 3, http://links.lww.com/INF/E927) revealed decreased precision with increased misclassification, with the association between past measles and lack of seroprotection against tetanus remaining statistically significant.

## DISCUSSION

Measles was associated with subprotective tetanus antibody in a sample of children in the DRC who were 9–59 months of age and fully vaccinated with the DTwPHibHepB vaccine. Measles and wealth index were the variables most consistently associated with changes in tetanus antibody, with both variables statistically significant in the linear and logistic regressions, as well as the GMC mean difference comparison. Previous work^31,32^ has shown that measles virus infection can reduce previously acquired humoral immunity, and our findings of decreased tetanus antibody and decreased tetanus seroprotection associated with measles disease agree with what is known of measles-associated immune amnesia. Wealth index, specifically residing in a household from the poorest wealth quintile, is also a known predictor of decreased tetanus seroprotection,^12^ possibly due to reduced access to health care.

The more than 2-fold increase in odds of tetanus toxoid seroprotection among children vaccinated against measles warrants further comment. Others have found vaccination against measles virus to be associated with reduction in all-cause mortality in children less than 5 years of age, in both resource-poor and -wealthy countries.^33–35^ Further, improvement in child proliferative response to tetanus toxoid antigen after receipt of a second measles-mumps-rubella vaccine has been observed.^36^ Although our study did not examine measles-mumps-rubella vaccine and our measurements were limited to tetanus IgG antibody and therefore not comparable with this previous work, there may be value in future work examining changes in humoral immune response against tetanus toxoid following measles vaccination.

According to our examination of measles AR stratified by age, the ARv did not differ greatly from our previous^22^ measles serologic study utilizing a larger sample of children 9–59 months of age participating in the 2013–2014 DRC DHS. For instance, in the 9–11 months of age group in our current study, the AR_V_ was 2.0, compared with an AR_V_ of 1.6 in the previous larger measles serologic study. In the 12–23 months of age group in our current study, the AR_V_ was 1.9, compared with an AR_V_ of 0.9 in the larger serologic study. In the 24–59 months of age group in our current analysis, the AR_V_ was 8.7, compared with 7.4 in the larger serologic study. AR_U_ findings in this study were less consistent with our previous measles serologic study, with the 9–11, 12–23 and 24–59 months of age groups showing AR_U_ results of 0 versus 4.0, 0 versus 7.1 and 20.0 versus 12.9, in the current study versus previous work, respectively.

The AR_V_ of 8.6 among the vaccinated 24–59-month-old children should be understood in light of the age at time of measles vaccination among measles cases, as 32 of these 34 children (94%) received measles vaccination at less than 12 months of age. Other work has shown similar AR_V_ in measles outbreak situations, with a reported outbreak in the United States in the 1970s showing a 6.3 AR_V_ among children vaccinated at less than 12 months of age, versus an AR_V_ of 1.7 among those vaccinated at 12 months of age or older, and an AR_U_ of 8.5.^37^ It must be noted that the percentage of children 24–59 months old and vaccinated at less than 12 months of age yet who were not measles cases was still high (308/359, 86%). While misclassification of measles cases is certainly a concern given our inability to obtain laboratory confirmation of IgM antibody, it is also important to consider that our sample for this analysis is more highly vaccinated, wealthier, and more urban than in the previously mentioned studies utilizing DHS DRC data. Since these variables can impact access to health services and so could impact health outcomes, interpretation of findings should be done with these characteristics of our sample in mind.

Previous work suggests that malnutrition does not suppress immunologic response to tetanus vaccination,^38,39^ likely due to vaccination-induced immunity to tetanus being mediated via the humoral immune system, while malnutrition primarily depresses cell-mediated immunity,^40^ although there have been findings of reduced IgG tetanus antibody (as measured via enzyme-linked immunosorbent assay) in stunted children compared with controls of normal nutritional status.^41^ Our study showed a statistically significant mean difference in GMC among severely stunted children versus all others, as well as a negative association with seroprotective tetanus antibody levels in the logistic regression model. While it is not possible to know whether severe stunting at the time of the DHS would have been ongoing at the time of vaccination, due to the chronic nature of stunting, it is plausible that children who were severely stunted at the time of the DHS might also have been severely stunted at the time of tetanus vaccination.

To the authors’ knowledge, this is the first study examining an association between past measles cases and decreased vaccine-induced tetanus antibody and is in agreement with recent work demonstrating decreases in previously acquired immunity.^31^ Strengths of this study include a large, nationally representative sample, availability of dated card vaccination reports for both measles and tetanus vaccinations, and increased confidence in maternal measles reports due to the availability of measles serologic data. Challenges include, as described in previous work,^7^ lack of laboratory-confirmed diagnosis of measles. However, other work utilizing household surveys has shown that maternal report of childhood illness can be a valid measure,^42^ and we found that most maternally reported measles cases in our study met the LOQ serologic inclusion criterion. Even so, we cannot be certain that maternally reported measles cases meeting LOQ criterion are correctly classified without laboratory confirmation. As we explored this limitation further, we found that the GMC of children with no maternal report of measles and no measles vaccination, although present, was not seroprotective, and also found that there was a statistically significant mean difference of GMC between unvaccinated children with versus without maternal report of measles. Finally, we found that potential increased misclassification of measles cases among vaccinated children would likely decrease precision of our estimates, but we were not seeing consistent bias away from the null. While these analyses do not completely mitigate the lack of laboratory-confirmed measles data, they provide increased confidence in our results.

Children with dated card report were more likely to be from urban areas, wealthier, and of better nutritional status than the entire group of DHS participants 9–59 months of age. While this may mean that an association between past measles cases and tetanus immunity could be different among poorer, more rural and less nourished children in the DRC, it is also possible that such an association would be more severe than what is seen in this study due to the additional health disparities that these children face.

These findings suggest the need for additional laboratory studies examining measles’ impact on preexisting, vaccine-induced immunity and underscore the need for continued evaluation and improvement of DRCs measles vaccination program.

## CONCLUSIONS

This study identifies an association with prior measles and decreased and subprotective levels of tetanus antibody among children in the DRC who were fully vaccinated for tetanus. Additional research should continue to examine the impact of measles on immunity to tetanus in this and other resource-poor populations. Perseverance in both understanding immunity and immunologic response to vaccine-preventable diseases, such tetanus and measles, as well as narrowing existing gaps in immunization programs, is needed to effectively improve the health of children in resource-limited countries such as the DRC.

## Supplementary Material


