# Supplementary material for: Reported History of Measles and Long-term Impact on Tetanus Antibody Detected in Children 9–59 Months of Age and Receiving 3 Doses of Tetanus Vaccine in the Democratic Republic of the Congo
Source: Pediatr Infect Dis J. 2023 Feb 9;42(4):338–45. doi: 10.1097/INF.0000000000003840 (PMC9990594; doi:10.1097/INF.0000000000003840)
Supplement: Supplementary file 1 [file inf-42-338-s001.docx]

| **Supplementary Table 1**: Geometric mean concentrations (GMC) of tetanus antibody IU among children 9-59 months of age.^12^ | | | | | | | |
| --- | --- | --- | --- | --- | --- | --- | --- |
|  | **n** | **GMC and 95% CI** | | | | **Mean Difference and Standard Error** | **P-value** |
|  |  |  |  |  |  |  |  |
|  |  | **Measles -** | | **Measles +** | |  |  |
| **Age in months** |  | **n** |  | **n** |  |  |  |
| 9-11 | 75 | 74 | 0.245 (0.157-0.384) | 1 | **0.155** **(---)** | **-0.459 (0.228)** | **0.0442** |
| 12-23 | 233 | 229 | 0.315 (0.256-0.387) | 4 | 0.404 (0.101-1.608) | 0.249 (0.686) | 0.717 |
| 24-35 | 179 | 172 | 0.259 (0.209-0.321) | 7 | 0.212 (0.149-0.300) | -0.199 (0.199) | 0.3163 |
| 36-47 | 105 | 90 | 0.253 (0.188-0.340) | 16 | **0.106** (0.084-0.134) | **-0.868 (0.183)** | **<.0001** |
| 48-59 | 119 | 105 | 0.209 (0.160-0.274) | 14 | **0.149** (0.061-0.363) | -0.343 (0.448) | 0.4447 |
| **Total** | 711 | 669 | 0.265 (0.228-0.309) | 42 | **0.155** (0.103-0.232) | **-0.540 (0.201)** | **0.0074** |
|  |  |  |  |  |  |  |  |
| **Measles vaccination status** |  | **Vaccinated** | | **Unvaccinated** | |  |  |
|  |  | 649 | 0.262 (0.226-0.302) | 63 | 0.213 (0.131-0.345) | 0.207 (0.239) | 0.386 |
|  |  |  |  |  |  |  |  |
| **Malaria status3** |  | **Malaria -** | | **Malaria +** | |  |  |
|  |  | 630 | 0.263 (0.225-0.307) | 82 | 0.216 (0.170-0.276) | -0.194 (0.123) | 0.115 |
|  |  |  |  |  |  |  |  |
| **Residence** |  | **Urban** | | **Rural** | |  |  |
|  |  | 347 | 0.303 (0.251-0.366) | 365 | 0.219 (0.175-0.274) | **-0.324 (0.149)** | **0.03** |
|  |  |  |  |  |  |  |  |
| **Maternal education** |  | **≥ 7 years education** | | **< 7 years education** | |  |  |
|  |  | 360 | 0.298 (0.250-0.357) | 352 | 0.220 (0.179-0.271) | **0.303 (0.129)** | **0.019** |
|  |  |  |  |  |  |  |  |
| **Severe stunting** |  | **Severely stunted -** | | **Severely stunted +** | |  |  |
|  |  | 579 | 0.274 (0.233-0.322) | 133 | **0.195** (0.156-0.242) | **-0.341 (0.119)** | **0.0042** |
|  |  |  |  |  |  |  |  |
| **Wealth index** |  | **Wealthier** | | **Poorest** | |  |  |
|  |  | 636 | 0.272 (0.234-0.317) | 75 | **0.156** (0.119-0.205) | **-0.555 (0.154)** | **0.0003** |
| **Grand Total** | **711** | **0.257 (0.221-0.298)** | | | | **---** | **---** |
| ^1^Bolded geometric mean estimates (excluding "Total" row) indicate that antibody was below seroprotective cutoff. | | | | | |  |  |
| ^2^Bolded 95% confidence intervals indicate statistically significant estimates. | | | | | |  |  |
