# Supplementary material for: Reported History of Measles and Long-term Impact on Tetanus Antibody Detected in Children 9–59 Months of Age and Receiving 3 Doses of Tetanus Vaccine in the Democratic Republic of the Congo
Source: Pediatr Infect Dis J. 2023 Feb 9;42(4):338–45. doi: 10.1097/INF.0000000000003840 (PMC9990594; doi:10.1097/INF.0000000000003840)
Supplement: Supplementary file 2 [file inf-42-338-s002.docx]

| **Supplementary Table 2**. Examination of Limit of Quantitation (LOQ), maternal report of measles, measles seroprotection status, and Geometric Mean Concentration (GMC) of tetanus antibody within the following four groups of children: measles-unvaccinated + no maternal measles report, measles-unvaccinated + maternal measles report, measles-vaccinated + no maternal measles report, measles-vaccinated + maternal measles report. | | | | | | | | | | | | | |
| --- | --- | --- | --- | --- | --- | --- | --- | --- | --- | --- | --- | --- | --- |
| **Measles-unvaccinated children** | | | | | | | | | | | | | |
| **Age** | **Total** | **Children with no measles vx, no maternal report** | **Children meeting LOQ^1^ criterion (%)** | **Children seroprotected against measles (%)** | **GMC^2^** | **GMC 95%CI** | **Children with no measles vx, maternal measles report** | **Children meeting LOQ criterion (%)** | **Children seroprotected against measles (%)** | **GMC** | **GMC 95%CI** | **Mean difference^3^** | **95%CI^4^** |
| **9-11 months** | **26** | 26 | 8 (29) | 5 (20) | 0.031 | 0.016, 0.0600 | 0 | --- | --- | --- | --- |  |  |
| **12-23 months** | **27** | 27 | 14 (52) | 10 (37) | 0.061 | 0.025, 0.150 | 0 | --- | --- | --- | --- |  |  |
| **24-35 months** | **4** | 4 | 3 (93) | 2 (44) | 0.071 | 0.034, 0.146 | 0 | --- | --- | --- | --- |  |  |
| **36-47 months** | **4** | 2 | 1 (77) | 1 (77) | 0.087 | 0.01, 0.763 | 2 | 2 (100) | 2 (100) | 2.046 | 1.602, 2.612 |  |  |
| **48-59 months** | **3** | 3 | 3 (100) | 3 (100) | 1.358 | 0.847, 2.178 | 0 | --- | --- | --- | --- |  |  |
| **Total** | **63** | **62** | **29 (48)** | **21 (34)** | **0.054** | **0.03, 0.0956** | **2** | **2 (100)** | **2 (100)** | **2.046** | 1.602, 2.612 | **3.644** | **2.982, 4.307** |
|  |  |  |  |  |  |  |  |  |  |  |  |  |  |
| **Measles-vaccinated children** | |  |  |  |  |  |  |  |  |  |  |  |  |
| **Age** | **Total** | **Children with measles vx, no maternal report** | **Children meeting LOQ criterion (%)** | **Children seroprotected against measles (%)** | **GMC** | **GMC 95%CI** | **Children with measles vx, maternal measles report** | **Children meeting LOQ criterion (%)** | **Children seroprotected against measles (%)** | **GMC** | **GMC 95%CI** | **Mean difference** | **95%CI^4^** |
| **9-11 months** | **49** | 48 | 32 (67) | 24 (51) | 0.078 | 0.0312, 0.193 | 1 | 1 (100) | 1 (100) | 0.386 | --- |  |  |
| **12-23 months** | **207** | 202 | 179 (89) | 141 (70) | 0.199 | 0.155, 0.256 | 4 | 4 (100) | 4 (100) | 0.316 | 0.229, 0.438 |  |  |
| **24-35 months** | **176** | 167 | 153 (91) | 139 (83) | 0.260 | 0.175, 0.387 | 8 | 7 (81) | 7 (79) | 0.246 | 0.037, 1.652 |  |  |
| **36-47 months** | **101** | 88 | 82 (93) | 76 (86) | 0.386 | 0.289, 0.516 | 13 | 13 (100) | 13 (100) | 0.603 | 0.368, 0.987 |  |  |
| **48-59 months** | **116** | 102 | 101 (99) | 89 (87) | 0.377 | 0.294, 0.482 | 14 | 14 (100) | 13 (89) | 0.247 | 0.133, 0.459 |  |  |
| **Total** | **649** | **607** | **548 (90)** | **469 (77)** | **0.244** | **0.204, 0.292** | **42** | **40 (96)** | **38 (91)** | **0.341** | **0.201, 0.579** | 0.336 | -0.180, 0.853 |
| ^1^Limit of Quantitation | | | | | |  |  |  |  |  |  |  |  |
| ^2^Geometric Mean Concentration | | | | | |  |  |  |  |  |  |  |  |
| ^3^Comparing measles-unvaccinated children, with and without maternal report | | | | | |  |  |  |  |  |  |  |  |
| ^4^Bolded 95% confidence intervals indicate statistically significant estimates | | | | | |  |  |  |  |  |  |  |  |
