# Supplementary material for: Reported History of Measles and Long-term Impact on Tetanus Antibody Detected in Children 9–59 Months of Age and Receiving 3 Doses of Tetanus Vaccine in the Democratic Republic of the Congo
Source: Pediatr Infect Dis J. 2023 Feb 9;42(4):338–45. doi: 10.1097/INF.0000000000003840 (PMC9990594; doi:10.1097/INF.0000000000003840)
Supplement: Supplementary file 3 [file inf-42-338-s003.docx]

|  | **Supplementary Table 3**. Examination of exposure misclassification by measles vaccination status^1^. | | | | | | | | |
| --- | --- | --- | --- | --- | --- | --- | --- | --- | --- |
| a. | **Original measles classification** | | | | c. | **Considering 25% of measles cases among vaccinated children to be false positives and re-classifying as measles non-cases** | | | |
|  |  | **Sub-seroprotection^2^** | **Seroprotection** | ***Total*** |  |  | **Sub-seroprotection** | **Seroprotection** | *Total* |
|  | **Measles case** | **26** | **7** | ***33*** |  | **Measles case** | **20** | **5** | ***25*** |
|  | Vaccinated against measles | *24* | *7* | *31* |  | Vaccinated against measles | *18* | *5* | *23* |
|  | Unvaccinated | *2* | *0* | *2* |  | Unvaccinated | *2* | *0* | *2* |
|  | **Measles non-case** | **267** | **343** | ***610*** |  | **Measles non-case** | **273** | **345** | ***618*** |
|  | Vaccinated against measles | *238* | *318* | *556* |  | Vaccinated against measles | *244* | *320* | *564* |
|  | Unvaccinated | *29* | *25* | *54* |  | Unvaccinated | *29* | *25* | *54* |
|  |  |  |  |  |  |  |  |  |  |
|  | ***Total*** | ***293*** | ***350*** | **643** |  | ***Total*** | ***293*** | ***350*** | **643** |
|  | **OR (95%CI): 0.21 (0.09, 0.49)^3^** | |  |  |  | **OR (95%CI): 0.198 (0.073, 0.534)** | |  |  |
|  |  |  |  |  |  |  |  |  |  |
| b. | **Considering 10% of measles cases among vaccinated children to be false positives and re-classifying as measles non-cases** | | | | d. | **Considering 50% of measles cases among vaccinated children to be false positives and re-classifying as measles non-cases** | | | |
|  |  | **Sub-seroprotection** | **Seroprotection** | ***Total*** |  |  | **Sub-seroprotection** | **Seroprotection** | ***Total*** |
|  | **Measles case** | **24** | **6** | ***30*** |  | **Measles case** | **14** | **4** | ***18*** |
|  | Vaccinated against measles | *22* | *6* | *28* |  | Vaccinated against measles | *12* | *4* | *16* |
|  | Unvaccinated | *2* | *0* | *2* |  | Unvaccinated | *2* | *0* | *2* |
|  | **Measles non-case** | **269** | **344** | ***613*** |  | **Measles non-case** | **279** | **347** | ***626*** |
|  | Vaccinated against measles | *240* | *319* | *559* |  | Vaccinated against measles | *250* | *322* | *572* |
|  | Unvaccinated | *29* | *25* | *54* |  | Unvaccinated | *29* | *25* | *54* |
|  |  |  |  |  |  |  |  |  |  |
|  | ***Total*** | ***293*** | ***350*** | **643** |  | ***Total*** | ***293*** | ***350*** | **643** |
|  | **OR (95%CI): 0.195 (0.079, 0.485)** | |  |  |  | **OR (95%CI): 0.230 (0.075, 0.708)** | |  |  |
|  | ^1^Data are unweighted. |  |  |  |  |  |  |  |  |
|  | ^2^Seroprotection against tetanus | |  |  |  |  |  |  |  |
|  | ^3^Calculated using SAS 9.4 with 95% Wald Confidence Limits | | |  |  |  |  |  |  |
